# Association between Cockroach-specific Immunoglobulin E and periodontitis in Korean male adults Based on Korean National Health and Nutrition Examination Survey

**DOI:** 10.1038/srep46373

**Published:** 2017-04-12

**Authors:** Mihee Hong, Jun-Beom Park, Young Soo Kim, Dong-Hee Lee, HeeYeon Kim, Jae-Im Lee, Hyo-Suk Ahn, Tae Seo Sohn, Tae-Kyu Lee, Jae Yen Song, Seong Cheol Jeong, Chang Dong Yeo, Hiun Suk Chae, Kyung Do Han, David Vu, Young Bok Lee

**Affiliations:** 1 Epidemiology Study Cluster of Uijeongbu St. Mary’s Hospital, Uijeongbu St. Mary’s Hospital, College of Medicine, The Catholic University of Korea, Seoul, South Korea; 2 Department of Periodontics, Seoul St Mary’s Hospital, College of Medicine, The Catholic University of Korea, Seoul, Korea; 3 Department of Biostatistics, College of Medicine, The Catholic University of Korea, Seoul, Korea; 4 Wing Dental Center, Alberta, Canada

## Abstract

Periodontitis is an inflammatory disease affecting the tooth supporting tissues (periodontium) and associated with chronic diseases such as cardiovascular disease and insulin resistance. However, there has been no nation-wide population based epidemiologic study regarding any association between periodontitis and serum IgE. Among the 8,958 participants in the 2010 Korean National Health and Nutrition Examination Survey (KNHANES V-1), 1,731 adults aged 19 to 64 who had measured serum IgE were included in the analysis. Dentists examined the periodontal status of the participants. Multiple logistic regression analyses were used to evaluate the odds ratio of periodontitis in association with total IgE and specific IgE to cockroach and house dust mite. In males, total IgE showed a positive correlation with the presence of periodontitis. The participants in the highest tertile of cockroach specific IgE (T3, >31.6 kU/L) had a significantly increased risk of periodontitis (OR = 2.108; 95% CI, 1.233–3.606). In females, the inverse association occurred between total IgE and periodontitis (OR = 0.409; 95% CI, 0.200–0.839). The present study firstly demonstrated the association between periodontitis and serum IgE, using the Korean nationwide, population-based, cross-sectional health examination and survey. This study suggested a positive correlation between periodontitis and cockroach-specific IgE in Korean male adults.

Periodontitis is a common chronic inflammatory disease and a major cause of tooth loss^1^. Periodontitis is characterized by the destruction of periodontium following an inflammatory host response secondary to infection by periodontal bacteria. Pathogenic microorganisms, genetic and environmental factors, especially tobacco use, contribute to the major risk factors of periodontitis^1^. Recently, it has been reported that bacterial biofilm plays an important role in the onset and subsequent development of periodontitis, participating in the formation of periodontal pockets, destruction of connective tissue, and resorption of alveolar bone by means of an immune-pathogenic mechanism^2^,^3^. There are hundreds of species that reside naturally in the oral cavity, but only a small number has been associated with the progression of disease and considered to be possible pathogens in periodontitis; *Porphyromonas gingivalis, Aggregatibacter Actinomycetemcomitans, Capnocytophaga spp, Eikenella corrodens, Prevotella intermedia, Campylobacter rectus, Fusobacterium nucleatum,* and *Treponema denticola*^4^.

Periodontitis has been considered to be associated with chronic diseases. Several studies have revealed the association of periodontitis with insulin resistance^5^,^6^, cardiovascular disease^7^, gastric *Helicobacter pylori* infection^8^ and liver fibrosis in patients with hepatitis virus infection^9^. The presence of periodontitis is considered a risk factor for these chronic diseases. Schenkein *et al*. reported that oral immunization by bacterial antigens may account for systemic immune response and oral colonization lead to systemic inflammations^10^. Konig *et al*. reported that periodontal pathogen *A. Actinomycetemcomitans* is a candidate bacterial trigger of rheumatoid arthritis^11^.

Allergic diseases have been revealed to have an inverse association with periodontitis. Friedrich *et al*. reported that there was a negative association between periodontitis and respiratory allergies^12^,^13^. In Freidrich’s study (2006), sensitization to house dust mites was inversely associated with the presence of periodontitis^13^. These results supported the hygiene hypothesis of allergic disease that supports the protective effects of oral pathogens on the development of allergic diseases^14^. Hygiene hypothesis was originally proposed by Strachan who observed an inverse relationship between family size and the prevalence of hay fever^15^. According to hygiene hypothesis, a person who lacks of early childhood exposure to infectious agents is susceptible to allergic diseases. Early-childhood exposures to microbes prevent a shifting from a Th1 to a Th2 phenotype. Th2 cytokines are involved in immune responses to allergic disease, whereas Th1 cytokines are involved in cellular immune disease.

There are several reports that supports hygiene hypothesis of periodontitis to allergic disease. The detection of serum antibodies to oral pathogens in early childhood has provided evidence of an early immune response to these bacteria, and has shown a protective effect over allergic diseases^16^. Grossi *et al*. reported an inverse relationship between periodontal disease and an allergy-related outcome^17^; hay fever was significantly and inversely associated to periodontal disease severity. Arbes *et al*. reported associations between serum Immunoglobulin G (IgG) antibody levels to *A. actinomycetemcomitans* and *P. gingivalis* and subject-reported asthma, wheezing, and hay fever among the US population aged 12 years and older. The higher concentrations of IgG antibody to *P. gingivalis* were significantly associated with the lower prevalence of asthma, wheeze, and hay fever. Higher concentrations of IgG to *A. actinomycetemcomitans* were significantly associated with a lower prevalence of wheeze^18^. However, in a recent study, Gomes-Filho proposed a detrimental effect of periodontitis on asthma that opposed the hygiene hypothesis^19^.

In this study, we aimed to evaluate the association between periodontitis and the level of serum Immunoglobulin E (IgE) in general population. IgE is associated mainly with allergic reactions. An allergen-specific IgE test is done to check whether a person is allergic to a particular substance. In Korea, house dust mite, cockroach, mugwort, oak, Japanese hop, ragweed, and dog dander are important inhalant allergens^20^. House dust mite is the primary inhalant allergen^21^, and cockroach is the second leading allergen. The Korean National Health and Nutrition Examination Survey (KNHANES V-1) performed measurements of total IgE, house dust mite-specific IgE, and cockroach specific IgE in randomly selected participants. Using the population based database, we performed the statistical analysis that evaluate the association between periodontitis and serum IgE (total IgE, cockroach-specific IgE, and house dust mite-specific IgE).

## Results

### Demographics

The characteristics of the study participants are summarized in Table 1. Among the 1,731 participants, 383 participants were classified as having periodontitis group. Compared to the control group, the periodontitis group had a higher percentage of current smokers (34.3 ± 2.6%, P < 0.0001), and higher percentage of those who drink more than once a month (69.8 ± 2.7%, P < 0.0085). There was no statistical significance in exercise and income.

The periodontitis group presented the higher percentage of ‘once or less tooth brushing times per day’, and a lower percentage of ‘three or more brushing times per day’ when compared to the control group. A remaining tooth count of more than 28 was significantly lower in the periodontitis group.

The mean age of the periodontitis group (48.1 ± 0.6 years) was significantly higher than that of the control group (38.3 ± 0.4 years, P < 0.0001). In the periodontitis group, BMI (24.3 ± 0.2 kg/m^2^) and waist circumferences (83.5 ± 0.6 cm) were significantly higher than the control group (23.4 ± 0.1 kg/m^2^ and 79.4 ± 0.4 cm, respectively). The difference in white blood cell counts was statistically significant between the periodontitis group (6.2 × 10^3^/*μ*ℓ, 95% CI, 6.0–6.5) and the control group (6.0 × 10^3^/*μ*ℓ, 95% CI, 5.9–6.1).

### Association between sensitization to specific allergen and periodontitis

Because levels of IgE showed wide ranges (0–5000 kU/L), comparing the mean value of IgE does not seem to be appropriate to find out the association between levels of IgE and periodontis. Subjects were categorized into three groups according to the levels of total serum IgE, house dust mite-specific IgE, and cockroach-specific IgE.

In female, the cut-off levels between Tertile1 (T1) and Tertile2 (T2), and between Tertile2 (T2) and Tertile3 (T3) were 31.6 kU/L and 94.9 kU/L for total IgE; 0.03 kU/L and 0.09 kU/L for cockroach-specific IgE; and 0.03 kU/L and 0.3 kU/L for house dust mite-specific IgE, respectively. In male, the cut-off levels between Tertile1 (T1) and Tertile2 (T2), and between Tertile2 (T2) and Tertile3 (T3) were 67.3 kU/L and 269 kU/L for total IgE; 0.05 kU/L and 0.26 kU/L for cockroach-specific IgE; and 0.09 kU/L and 1.06 kU/L for house dust mite-specific IgE, respectively. The results of the logistic regression analyses between periodontitis and allergic sensitization were shown in Table 2.

In males, total IgE and allergic sensitization to the cockroach allergen showed a positive correlation with periodontitis. In model 1, the ORs of periodontitis in the middle Tertile (T2, total serum IgE; ≥67.3 kU/L and <269 kU/L) of total IgE was 2.067 (95% CI 1.269–3.367) compared to T1 (total serum IgE <67.3 kU/L). After adjusting for age, BMI, smoking and alcohol consumption status, exercise, income, education, metabolic syndrome, and white blood cell count, the OR in model 2 was 1.999 (95% CI 1.163–3.435) in males compared to total serum IgE (T1) in males. The highest tertile (T3) of cockroach-specific IgE in male showed increased ORs of 1.75 (95% CI, 1.086–2.821), 2.038 (95% CI, 1.212–3.428), and 2.108 (95% CI 1.233–3.606) respectively in three models of males. However, there was no association shown between periodontitis and house dust mite sensitization in males.

Conversely, in females, inverse associations between total serum IgE and periodontitis were shown (Table 2). In female adults, the highest tertile (T3, total IgE >94.9 kU/L) showed a significantly lower risk of periodontitis after adjusting the confounders in model 2 (OR = 0.449, 95% CI 0.225–0.897) and model 3 (OR = 0.409, 95% CI 0.200–0.839). Analysis showed no statistical significance between specific IgE and periodontitis in females.

## Discussion

To determine the effect of the hygiene hypothesis in Korean adults, we used the population-based database, KNHANES V-1. This study presented the association between both total IgE and specific IgE, and periodontitis in adult groups. The periodontitis group was significantly related to smoking, alcohol consumption, lower educational status, smaller numbers of remaining teeth, lower tooth brushing frequency, older age, BMI, waist circumferences, and metabolic syndrome (Table 1). The well-known associated risk factors of periodontitis were adjusted in logistic regression analysis. In the present study, the hygiene hypothesis was consistent in female adults. The highest total IgE group in females (T3, total IgE >94.9 kU/L) presented an inverse correlation with periodontitis. The cockroach-specific IgE and house dust mite-specific IgE showed negatively association with periodontitis; however, there was no statistically significance.

Unexpectedly, in males, the second tertile group of total IgE presented positive correlations with periodontitis in models 1 and 2 (Table 2). Additionally, higher allergic sensitization to cockroach allergen showed an increased risk for periodontitis, even after adjustments were made for age, BMI, smoking and alcohol consumption status, exercise, income, education level, metabolic syndrome, white blood cell count, frequencies of tooth brushing, and remaining teeth number status. The house-dust specific IgE was not associated with periodontitis in male adults. However, the cockroach-specific IgE was significantly associated with periodontitis in male adults.

The gender difference in results was suspected that men had higher smoking rate than women and seemed to be less hygienic than women. Another explanation is that men had higher level of total and allergen specific IgE than women (the cut-off levels of total IgE between Tertile1 (T1) and Tertile2 (T2), and between Tertile2 (T2) and Tertile3 (T3) were 31.6 kU/L and 94.9 kU/L in female, and 67.3 kU/L and 269 kU/L in male). However, the further studies of gender differences in association between serum IgE and periodontitis are needed to be evaluated.

The discrepancy between house dust mite allergen and cockroach allergen might be explained by the specific reaction to cockroach allergen. Cockroach allergen is known to increase inflammatory cytokines, such as IL-8, MCP-1, CCL20, and GM-CSF^22^, which result in epithelial damage on bronchial cells. Both house dust mite and cockroach allergens are known to induce a type 2 cytokine profile and mixed granulocytic inflammation in the airway. However, unlike house dust mite, cockroach-induced inflammation has shown to activate IL-6 trans-signaling and production of IL-17A by γ δT cells^23^. Previous research reported that cockroach extracts contain pepstatins, A-sensitive proteases that initiate the action of protease activated receptors (PAR)-2, and induce activation and degranulation of human eosinophils^24^. Wada *et al*. suggested two pathologic mechanisms of cockroach allergen: serving as allergenic proteins and stimulating inflammatory cells through proteolytic activities^24^. Page *et al*. demonstrated linkage of cockroach proteases to the activation of a variety of cells involved in the process of allergic airway inflammation, and in the role of PAR-2^25^.

The pro-inflammatory mechanism of cockroach allergen through PAR-2 is similar to the inflammatory reaction between *Porphyromonas gingivalis* and human gingival fibroblasts via PAR-2. Palm *et al*. revealed that the periodontal pathogen, *P. gingivalis,* facilitates periodontitis interaction with human gingival fibroblasts that activate PAR^26^. Although the sensitization to the cockroach does not imply direct interaction between the cockroach and human gingival fibroblasts, the inflammatory mechanisms of the cockroach allergen might play a role in inflammatory diseases, including periodontitis. Additional research is needed to identify mechanisms linking cockroach sensitization and periodontitis.

Another possible explanation can be that the cockroach-sensitized group might be different from the house dust mite-sensitized group in demographics. Several studies have demonstrated the geographical differences between house dust mite sensitization and cockroach sensitization^27^. Cockroach allergy is known to be associated with exposure in areas of heavy infestation^28^,^29^. Low socioeconomic status and race have been reported to be associated independently with sensitization to cockroach allergens. African American and Mexican American children had significantly higher odds of cockroach sensitivity than white children^30^. In this study, periodontitis group was significantly related to lower educational status and lower household income that reflect low socioeconomic status. Sensitization to cockroach is also assumed to be associated with low socioeconomic status in this study.

Our study did have several limitations. First, it was a cross-sectional study, which introduced difficulties in reasoning causality. Second, other specific allergens were not measured in this study. Third, the analysis of subgroups according to the specific IgE sensitization was not completed in this study. A difference between the house dust mite sensitized group and cockroach sensitized group was suspected. Fourth, gender differences were not further evaluated in this study. Fifth, the periodontitis definition (CPI) used in the present study does not take clinical attachment loss into consideration. Lastly, some response bias may have been present regarding the reporting of smoking and alcohol consumption status, since these data were collected through self-administered questionnaires.

Despite these limitations, to the best of our knowledge, this is the first population-based study to demonstrate the association between serum IgE and periodontitis. This study provided meaningful evidence for the positive correlation between cockroach-specific IgE and periodontitis in Korean adult males. The positive correlation between cockroach-specific IgE and periodontitis in Korean male adults was also firstly revealed in this study that opposed the hygiene hypothesis of periodontitis. Additional research is needed to identify mechanisms linking cockroach sensitization and periodontitis.

## Materials and Methods

### Study Population and Data Collection

This study analyzed the data from the 5th Korean National Health and Nutrition Examination Survey (KNHANES V-1), which was conducted from January 2010 to December 2010 by the Korea Centers for Disease Control and Prevention. The study design followed the tenets of the Declaration of Helsinki for biomedical research. The survey was conducted after approval from the Institutional Review Board of the Korea Centers for Disease Control and Prevention (IRB No. 2010–02CON-21-C). Survey participants included 77.5% of the selected sample of 10,938 subjects in 2010.

The study was conducted using a rolling sampling design that involved a complex, stratified, multistage, probability cluster survey of a representative sample of the civilian population of South Korea. A total of 192 sampling units were randomly selected from primary sampling units encompassing the target population. Each sampling unit contained 20 households; 3,800 households were surveyed in one year. The survey was conducted by four teams, and consisted of three parts: a health interview, a nutrition interview, and a health examination.

All questionnaires were administered either by the physicians or by trained interviewers in person at the participants’ homes. According to the National Health Enhancement Act, subjects had the right to refuse participation. All who agreed to take part provided written informed consent. The Korea Centers for Disease Control and Prevention obtained consent from the participants to draw blood during the health interview survey and use samples for further research.

**Figure 1:**
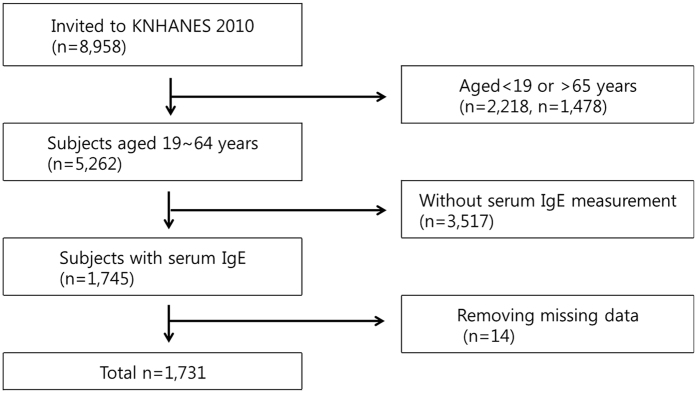
Flow diagram showing the selection of study participants.

Among 8,958 potential participants, those younger than 19 years (n = 2218) or older than 65 years (n = 1478) were excluded. In addition, 3,517 individuals were eliminated as participants due to lack of serum IgE measurements. After removing missing data (n = 14), the final study population was comprised of 1,731 participants with complete data sets (Fig. 1).

### Anthropometry and laboratory measurements

The health interviews and health behavior surveys included well-established questions to determine the demographic and socioeconomic characteristics of the subjects. The surveys included questions on age, sex, family income, education level, smoking and alcohol consumption status, exercise, and tooth brushing frequency. Smoking status was divided into three categories: current smoker, ex-smoker, or nonsmoker. Alcohol consumption status was categorized into no alcohol consumption and alcohol consumption more than once a month, and exercise status was divided into yes or no.

Blood pressure was measured twice on the right arm at a 5-minute interval using a standard mercury sphygmomanometer (Baumanometer; Baum, Copiague, NY, USA) and recorded as an averaged value. Blood samples were appropriately processed, immediately refrigerated, and transported in cold storage to the Central Testing Institute (Seoul, Korea). White blood cell counts were measured using laser low cytometry (XE-2100D, Sysmex, Kobe, Japan). Levels of glucose, high density lipoprotein cholesterol, and triglyceride were measured enzymatically using a Hitachi Automatic Analyzer 7600 (Hitachi, Japan).

Metabolic syndrome is defined as the presence of any 3 of 5 risk factors; (1) large waist circumference (≥90 cm for males, ≥80 cm for females); (2) elevated triglycerides (≥150 mg/dL) or drug treatment for elevated triglycerides; (3) reduced high density lipoprotein cholesterol (<40 mg/dL for males, <50 mg/dL for females) or drug treatment for reduced high density lipoprotein cholesterol; (4) elevated blood pressure (systolic ≥130 and/or diastolic ≥85 mm Hg) or antihypertensive drug treatment and; (5) elevated fasting glucose (≥100 mg/dL) or drug treatment for elevated glucose^31^.

### Measurement of serum IgE

The KNHANES randomly selected 12 people in one sampling unit by age (10 s, 20 s, 30 s, 40 s, 50 s, and over 60 s) and gender (male and female). A total 2,400 participants were chosen annually for IgE measurement. Total IgE and allergen-specific IgE were analyzed using a 1,470 WIZARD γ-Counter analyzer (PerkinElmer, Turku, Finland) with an immunoradiometric assay (ImmunoCAP 100, Phadia, Uppsala, Sweden). Two common indoor allergens were tested for house dust mite (*Dermatophagoides farinae*) and cockroach. We divided the subjects into three groups according to the serum levels: total IgE, cockroach-specific IgE, and house dust mite-specific IgE.

### Presence of periodontitis

To assess periodontal status, the World Health Organization (WHO) community periodontal index (CPI) was used. The codes and criteria for CPI scoring are as follows: normal (CPI = 0), gingival bleeding present (CPI = 1), calculus felt during probing (CPI = 2), a periodontal pocket of 4–5 mm (CPI = 3), and a pocket of >6 mm (CPI = 4). Periodontal pocket depths were measured at the mesiobuccal, mid-buccal, distobuccal, distolingual, midlingual and mesiolingual sites per tooth with a WHO periodontal probe. Periodontitis was defined as a CPI value of 3 or 4. Trained dentists examined the periodontal status of the participants. Training was provided to minimize errors in the periodontal measurements. In the 2010 KNHANES, 36 dentists performed periodontal examinations. For the measurements of CPI, simulated patients were used for the training. The trainings were performed from four to five times per dentist. Average Kappa value for the fourth quarter of 2010 was 0.611 (0.473–0.768).

### Statistical analysis

All continuous variables are presented as means and standard error. All categorical variables are presented as percentages. Logarithmic transformation was performed to achieve a normal distribution of the white blood cell count. Student *t*-tests or one-way analysis of variances were used to compare mean values for continuous variables; percentages were used for categorical variables, according to the presence of periodontitis. To estimate odds ratios (OR) of periodontitis according to total IgE levels and allergen-specific serum IgE levels, we conducted simple and multiple logistic regression analyses by using the generalized linear model for a complex survey design. The ORs and corresponding 95% confidence intervals (CIs) were calculated using a confounder adjustment for age (model 1); for age, BMI, waist circumference, smoking, alcohol consumption, exercise, income, education, white blood cell count (model 2); and for age, BMI, waist circumference, smoking, alcohol consumption, exercise, income, education, white blood cell count, frequencies of tooth-brushing, and self-reported oral status, number of remaining teeth (model 3). All analyses were performed using SAS (Statistical Analysis System, Version 9.3, SAS Institute Inc., Cary, NC, USA).

## Additional Information

**How to cite this article**: Hong, M. *et al*. Association between Cockroach-specific Immunoglobulin E and periodontitis in Korean male adults Based on Korean National Health and Nutrition Examination Survey. *Sci. Rep.* **7**, 46373; doi: 10.1038/srep46373 (2017).

**Publisher's note:** Springer Nature remains neutral with regard to jurisdictional claims in published maps and institutional affiliations.

## Figures and Tables

**Table 1:** Comparison of risk factors between no periodontitis group and periodontitis group.

Number of participants	No periodontitis	Periodontitis	P value
	1348	383	
Current smoker (yes, %)	22.8 ± 1.4	34.3 ± 2.6	0.0001
Alcohol consumption status (yes, %) (more than once a month)	61.5 ± 1.6	69.8 ± 2.7	0.0085
Exercise (yes, %)	24.1 ± 1.6	26.1 ± 3.0	0.5726
Income (Lowest Quartile, %)	11.4 ± 1.3	15.6 ± 2.4	0.1069
Education (>high school, %)	82.3 ± 1.2	64.4 ± 3.2	<0.0001
Tooth brushing (times per day)			<0.0001
Once or less (%)	9.5 ± 1.0	15.8 ± 2.5	
Twice (%)	46.7 ± 1.7	52.6 ± 3.1	
Three times or more (%)	43.8 ± 1.7	31.6 ± 2.8	
Self-reported oral status			<0.0001
Bad (%)	14.0 ± 1.2	8.7 ± 1.8	
Moderate (%)	47.7 ± 1.8	33.7 ± 3.6	
Good (%)	38.3 ± 1.6	57.6 ± 3.5	
Remaining teeth number of more than 28 (yes, %)	74.4 ± 1.4	49.7 ± 3	<0.0001
Age (years)	38.3 ± 0.4	48.1 ± 0.6	<0.0001
BMI (kg/m^2^)	23.4 ± 0.1	24.3 ± 0.2	0.002
Waist circumferences (cm)	79.4 ± 0.4	83.5 ± 0.6	<0.0001
Metabolic syndrome (%)	17.1 ± 1.4	33.9 ± 3.8	<0.0001
White blood cell (x 10^3^/*μ*ℓ) *	6.0 (5.9–6.1)	6.2 (6.0–6.5)	0.0412

Data presented as mean ± SE or % (SE). *Log transformation was applied to the value (95% CI).

**Table 2:** Regression analyses for dependent variable of periodontitis.

	Model 1		Model 2		Model 3	
	Male	Female	Male	Female	Male	Female
**Total IgE**						
T3	1.229(0.744,2.031)	0.622(0.343,1.13)	0.940(0.522,1.692)	**0.449(0.225,0.897)**	0.960(0.527,1.746)	**0.409(0.200,0.839)**
T2	**2.067(1.269,3.367)**	0.819(0.45,1.488)	**1.999(1.163,3.435)**	0.687(0.355,1.329)	1.786(0.992,3.217)	0.730(0.372,1.432)
T1	1	1	1	1	1	1
P value	0.0044	0.2924	0.0039	0.0762	0.0425	0.0495
**House dust mite**						
T3	1.215(0.731,2.022)	0.792(0.429,1.465)	1.105(0.640,1.909)	0.610(0.300,1.243)	1.111(0.644,1.916)	0.599(0.286,1.254)
T2	1.271(0.76,2.125)	0.674(0.364,1.248)	1.174(0.663,2.077)	0.587(0.294,1.173)	1.222(0.603,1.985)	0.587(0.295,1.166)
T1	1	1	1	1	1	1
P value	0.6364	0.4493	0.8595	0.2264	0.9131	0.2312
**Cockroach**						
T3	**1.75(1.086,2.821)**	1.006(0.564,1.797)	**2.038(1.212,3.428)**	0.851(0.439,1.649)	**2.108(1.233,3.606)**	0.875(0.445,1.723)
T2	1.512(0.974,2.345)	0.926(0.483,1.778)	1.323(0.817,2.143)	0.827(0.385,1.772)	1.341(0.823,2.186)	0.878(0.422,1.828)
T1	1	1	1	1	1	1
P value	0.0488	0.9654	0.0272	0.8358	0.0245	0.9022

Data are presented as odds ratio with 95% confidence interval. Model 1 : adjusted for age; Model 2: adjusted for age, BMI, *smoking*, alcohol consumption, exercise, income, education, metabolic syndrome, white blood cell count; Model 3 : adjusted for age, BMI, *smoking*, alcohol consumption, exercise, income, education, metabolic syndrome, white blood cell count, frequencies of tooth brushing, remaining teeth number status.
